# The mentalizing network updates neural representations of romantic interest in response to social feedback

**DOI:** 10.1093/scan/nsag074

**Published:** 2026-09-04

**Authors:** Benjamin M Silver, Christopher Baldassano, Lila Davachi, Kevin N Ochsner

**Affiliations:** Department of Psychology, Columbia University, New York, NY 10027, United States; Department of Psychology, Columbia University, New York, NY 10027, United States; Department of Psychology, Columbia University, New York, NY 10027, United States; Department of Psychology, Columbia University, New York, NY 10027, United States

**Keywords:** mentalizing network, representational similarity analysis, romantic interest, impression updating, social feedback

## Abstract

When we think about the start of a successful close relationship, we often imagine two people independently evaluating each other. But what if how much we like someone depends on how much we think they like us? In the current study, we asked how social feedback changes how one thinks about potential romantic partners, as well as how often one thinks about them. We hypothesized that the mentalizing network would play a role in both of these processes. During a functional magnetic resonance imaging scan, participants watched dating profile videos of eight different target individuals, both before and after receiving social feedback from the target. We found that neural representations in the mentalizing network changed most in response to social feedback that was incongruent with one’s initial evaluation, likely because romantic interest in another person is at least partially based on what one believes that other person is thinking. In addition, during a resting-state scan, reactivation frequency in the temporoparietal junction increased in response to *congruent* feedback. Considered together, these results demonstrate that the desire to be aligned with another person motivates both how and how often one thinks about that person, thus highlighting the importance of others’ social evaluations in our own.

## Introduction

Imagine you are on a first date. How do you decide if you want to see this person again? The impression you form of your date will likely be influenced by what you perceive to be your date’s impression of *you*. In this way, impressions are both socially dependent and temporally extended. In other words, they are continuously updated based on what we think another person thinks about us. Our understanding of the neural mechanisms underlying this process is limited because most social neuroscience studies of impression formation treat it as an isolated process rather than one that incorporates, and is motivated by, socially relevant feedback. In the current study, we used an extended, feedback-driven social evaluation paradigm to investigate how neural representations of potential romantic partners change after receiving social feedback that reveals how that potential partner feels about you.

If an impression of a target is at least partially dependent on what one thinks the target thinks about us, then we might hypothesize that such impressions depend upon the mentalizing network, which is a collection of brain regions important for drawing inferences about others’ mental states—including likes and preferences—as well as enduring traits (Atique *et al*. 2011, Baetens *et al*. 2014, Sahi and Eisenberger 2021). Specifically, we hypothesized that the dmPFC and the TPJ would be most relevant to evaluating romantic interest, based on prior work that demonstrates that these regions are important for encoding and accessing social information about others (Mitchell *et al*. 2005, Denny *et al*. 2012, Satpute *et al*. 2014, Meyer *et al*. 2019, Wagner *et al*. 2019, Jimenez and Meyer 2024) and trying to understand the thoughts and beliefs of other people (Saxe and Kanwisher 2004, Van Overwalle and Baetens 2009, Tamir *et al*. 2016, Thornton *et al*. 2019). To test these hypotheses, we asked whether, in the mentalizing network and its subregions, multivoxel representations of potential romantic partners were organized according to one’s romantic interest.

Romantic interest—like any impression—is a temporally extended process. However, social neuroscience studies investigating how impressions may change as we learn more about a target typically focus on whether or not dimensions of person perception, such as morality and competence, are consistently expressed (Cloutier *et al*. 2011, Ames and Fiske 2013, Mende-Siedlecki and Todorov 2016, Mende-Siedlecki 2018, Park and Young 2020). Although these studies implicate the dmPFC and the TPJ in impression updating about personality traits, whether or not they will also respond when impression updating is based on a target’s impression of us has not previously been studied.

In addition, impression updating studies typically only focus on the moment that an update occurs. In reality, there are two types of change that may occur *after* we receive social feedback in the affectively motivating context of romantic dating: *how* one thinks about someone and *how often* one thinks about them.

Changes in *how* one thinks about someone can be observed in changes in the structure of one’s multivoxel neural representations. For example, past work demonstrates between-subject differences in neural representations for the same social stimuli based on differences in social relationships (Parkinson *et al*. 2017, Guthrie *et al*. 2022) and political views (Leong *et al*. 2020, van Baar *et al*. 2021, Jacoby *et al*. 2024). In these studies, between-subject differences in perceptions of social stimuli were associated with differences in neural representations. These studies do not focus on within-subject differences, but the same principle can be applied: If someone’s perception of another person changes, their neural representations associated with that person should change as well. Thus, we might expect that neural representations of others will change more when targets provide social feedback that is unaligned with one’s initial impression.

Changes in *how often* one thinks about someone might also occur, since social neuroscience studies demonstrate that how often one thinks about something is indicative of their priorities (Gruber *et al*. 2016, Schapiro *et al*. 2018, Jimenez and Meyer 2024, Yu *et al*. 2024). These studies, which demonstrate reactivation effects both within and outside of the mentalizing network, led us to hypothesize that reactivation frequency will increase in response to social feedback (in general) due to the motivational relevance of the information, although it is unclear what *type* of social feedback participants will find most motivating. Participants may be motivated by the degree of feedback alignment, whereby aligned feedback speaks to our desire to connect with others, while unaligned feedback may serve as an expectancy violation (Somerville *et al*. 2010, Villano *et al*. 2020). Building on previous work in the memory literature (Gruber *et al*. 2016, Tambini and Davachi 2019, Yu *et al*. 2024), we also expect to see evidence of reactivation in the hippocampus. Although we do not necessarily expect this region to show a reactivation frequency increase in response to feedback, it is possible that unaligned feedback will be treated as an expectancy violation, which has been shown to increase hippocampal activity (Bein *et al*. 2023).

In summary, in the current study, we investigated the role of the mentalizing network in the formation and updating of romantic interest for potential romantic partners. We asked (i) whether patterns of activity in the mentalizing network are organized according to romantic interest, and, if they are, what information is used in that assessment, (ii) whether and how patterns of activity in the mentalizing network for potential romantic partners change in response to feedback about the partners’ own romantic interest, and (iii) whether and how this feedback might change the frequency with which neural representations of these potential partners might be reactivated in the mentalizing network. To investigate these questions, participants completed an fMRI scan while watching multiple dating profile videos for a series of targets, both before and after receiving social feedback from the targets. In addition, participants completed resting state scans after watching each set of dating profile videos.

## Materials and methods

### Participants

Participants were required to be between 18 and 29 years old to ensure that a large age difference between the participant and a potential romantic partner was not a factor in the participant’s romantic interest, and to be actively looking for a romantic partner via dating apps. Our final sample consisted of 30 total participants (age *M* = 23.50, SD = 2.67). Thirteen participants were men (8/13 were interested in women) and 17 were women (14/17 were interested in men). The breakdown of participants’ race was as follows: 9 Asian, 2 Black/African American, 2 White-Hispanic or Latino, 14 White-Not Hispanic or Latino, and 3 Other.

### Stimuli

During the fMRI scan, participants watched 90-s videos of potential romantic partners. Each video featured a person speaking directly to the camera about three different topics that one might find on a dating profile. The person in the video (the target) spoke about each topic for roughly 30 s. These videos were developed for this study with actors hired from the website Backstage. Each actor (eight men and eight women) made two videos. For a full description of how the videos were made, see Supplementary Material.

### Behavioral procedures

Upon arriving at the neuroimaging center for their fMRI scan, participants were told that they were going to have 10 min to make a 90-s dating profile video, in which they would respond to three prompts for approximately 30 s each. The resulting video was intended to be similar in format to the target videos that they would view during the scan. Participants were told that their video would be uploaded to a study database, and that upon uploading, a large network of “at-home” participants who had previously made similar videos would be pinged to view the actual participant’s video and indicate their perceived romantic interest in the actual participant. The actual participant was told that they would be “matched” with the first eight at-home participants to respond, and that during the study, they would get to watch and rate their romantic interest in these at-home participants. In addition, they were told that they would get to see whether or not the at-home participants were romantically interested in them in return.

In reality, the “at-home” participants in the videos were merely the actors described above and were not active participants in the study. The actual participant’s video was not actually uploaded to a database, and no other participants in the study viewed their video. The indications of (non-)reciprocal romantic interest that the participant saw during the scan were pseudorandom (see below). Participants were debriefed after completing the study, and were asked three questions to gauge their belief in the cover story: whether they wanted to receive the contact information of one of the targets, whether they wanted to message one of the targets, and—after being fully debriefed—whether they believed the cover story.

### Scanner procedures

**Figure 1:**
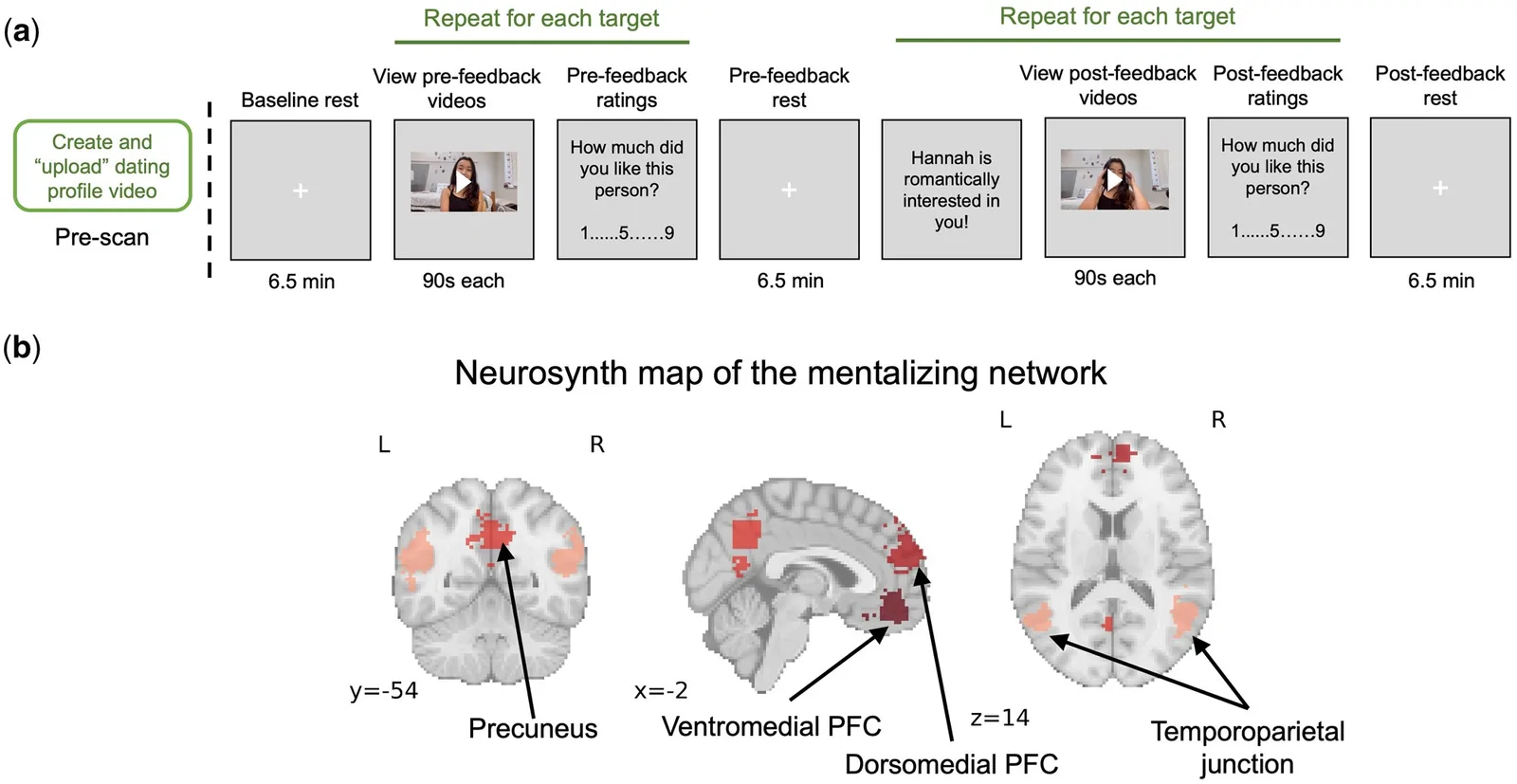
Paradigm and ROIs. (a) Participant procedures before and during the fMRI scan. Participants watched videos of eight different targets, both before and after receiving social feedback from the target. (b) Neurosynth map of the mentalizing network. Regions include the bilateral temporoparietal junction, bilateral temporal pole, precuneus, dorsomedial prefrontal cortex, and ventromedial prefrontal cortex. All analyses were first conducted on the mentalizing network as a whole. Follow-up analyses were conducted separately in each component region.

Participants began the neuroimaging session with a 6.5-min resting state scan with the instruction to keep their eyes open while looking at a white crosshair on a gray screen. Then, participants watched the first set of dating profile videos, hereafter called the pre-feedback videos. In a single run, participants watched one video of each of the eight targets, in a random order (Fig. 1a). After each video finished playing, there was a 2-s ITI, followed by five slider questions, presented one at a time. The five questions were as follows: “How romantically compatible do you believe you are with this person?” “How similar do you believe you are to this person?” “How physically attractive do you find this person?” “How interested were you in what this person was talking about?” “How much do you like this person?” Questions were answered on a continuous slider that was presented to participants as having minimum, midpoint, and maximum values of 1, 5, and 9. All five questions were highly correlated with each other and so were reduced to a single factor using an exploratory factor analysis. We term this factor romantic interest and use it in our behavioral analyses. Participants had 8 s to respond to each question; if the participant had not submitted an answer by the end of the 8 s, their mouse position along the slider was recorded (this occurred on 2.8% of trials across all participants). Following the slider questions, there was a 5-s ITI, and then participants viewed the next video.

Following the pre-feedback videos, participants completed another 6.5 resting state scan. Then, participants viewed a second set of dating profile videos for the same targets, hereafter called the post-feedback videos. (As a reminder, each target made two videos. The order of each target’s video presentation—whether it was in the pre-feedback videos or post-feedback videos—was counterbalanced between participants.) The post-feedback video-viewing was nearly identical to the pre-feedback video-viewing, except that each video presentation was immediately preceded by a feedback presentation. Participants were shown a photo of the target whose video they were about to view, along with one of two feedback messages: “[The target] said that [she/he] thinks she/he is romantically compatible with you” (positive feedback) or “[The target] said they [she/he] does not think that [she/he] is romantically compatible with you” (negative feedback). The feedback was pseudorandom via the following procedure: Participants’ initial compatibility ratings were binarized into positive ratings (5 or above) or negative ratings (below 5). For each category of targets (initial positive ratings and initial negative ratings), half of the targets provided positive feedback and half provided negative feedback, meaning that across all targets, half provided feedback that was aligned with the participant’s initial romantic interest (congruent feedback) and half provided feedback that was unaligned with the participant’s romantic interest (incongruent feedback). Thus, there were four feedback conditions: Positive-congruent, positive-incongruent, negative-congruent, and negative-incongruent. The feedback was presented for 5 s. Following a 2-s ITI, participants viewed that target’s post-feedback video.

After viewing the post-feedback videos, participants completed a third resting state scan. Following the scan, participants underwent a cover story debriefing in a testing room.

### fMRI scanning

Imaging data were acquired on a 3T Siemens scanner with a 64-channel head coil at the Zuckerman Mind Brain and Behavior Institute at Columbia University. Anatomical images were collected using a T1-weighted protocol (1 mm^3^ voxels, 176 slices per slab with 1 mm thickness, TR = 2300 ms, TE = 2.98 ms). Functional images were collected with a T2*-weighted gradient-echo EPI sequence (TR = 1000 ms; TE = 30; flip angle = 62°; 2.5 mm^3^ voxels), with 48 slices oriented parallel to the anterior commissure—posterior commissure (AC–PC) line. We also collected in-plane field map scans to improve co-registration between anatomical and functional images.

### fMRI preprocessing

Results included in this manuscript come from preprocessing performed using *fMRIPrep* 20.2.6 (Esteban *et al*. 2019). See Supplementary Methods for a full description of fMRIPrep preprocessing steps.

Our primary ROI was the entire mentalizing network, as defined by the mentalizing network map on Neurosynth (Yarkoni *et al*. 2011) and resampled to our data using AFNI’s 3dresample function (Cox 1996). The mentalizing map contained seven clusters that had over 100 voxels, which served as additional ROIs for follow-up analyses: bilateral temporoparietal junction, bilateral temporal pole, dmPFC, vmPFC, and the precuneus. Our hippocampal ROI was created using the Freesurfer output from fMRIPrep and the hippocampus was divided into anterior and posterior subregions by dividing the hippocampal ROI into thirds along the long axis of the hippocampus. The anterior third became our anterior hippocampus ROI, while the posterior two thirds became the posterior hippocampus ROI, in line with previous work that divides the hippocampus into anterior and posterior at the uncal apex (Thorp *et al*. 2022). Templates for neural representations of each target (see below) were created for each ROI.

### fMRI analyses

The majority of our functional imaging analyses were conducted by analyzing the pattern similarity between temporally averaged neural representations for each target. For each participant, for each video, we extracted a TR × voxel matrix of neural activity, where each TR was a timepoint during the video and each voxel was a location within our ROI. For each of these matrices, we averaged across all TRs to create a single, one-dimensional vector, where each point in the vector represents the average activity in a particular voxel across all TRs for that video. We did this separately for every participant, so that every participant had 16 neural pattern templates (one for each video they viewed). The majority of our analyses concerned Pearson correlations between these templates.

To determine if the mentalizing network and/or any of its subregions were tracking romantic interest for the targets, we ran a between-subjects representational similarity analysis (RSA) for each video in each ROI by correlating a neural dissimilarity matrix and a behavioral rating distance matrix. We then tested significance with a permutation test. Specifically, we generated 1000 permuted orders of our participants. We used each permutation to reorder the rows and columns of the neural dissimilarity matrix before correlating with the behavioral rating distance matrix, ensuring that there was no meaningful relationship between the two matrices. For each permutation, we calculated the average correlation across all videos so we could determine if the true average correlation was higher than the fifth percentile of our distribution of correlations. We repeated this analysis with variations of temporally averaged neural patterns, including temporally averaged patterns made up of just the first 10 TRs, as well as temporally averaged patterns with just the last 10 TRs, to determine if the brain’s tracking of romantic interest was stronger at the start or end of the video.

Next, we wanted to determine the impact of feedback on neural representations. For each participant, for each profile, we calculated the Pearson correlation between the profile’s pre-feedback neural pattern and the profile’s post-feedback neural pattern. We then ran three linear regressions to compare the strength of these pre-post correlations across three different pairs of conditions: positive vs negative feedback (feedback valence); congruent vs incongruent feedback (feedback congruence); and high vs low initial romantic interest (initial romantic interest). Feedback was considered congruent if the participant’s initial rating was below 5 AND the target feedback was negative, OR if the participant’s initial rating was 5 or above AND the target feedback was positive.

Our final suite of analyses concerned reactivation of profiles during post-encoding rest. For each temporally averaged neural pattern, we calculated the Pearson correlation between the temporally averaged pattern and the pattern of neural activity at each TR during the baseline resting state scan, before the participants had viewed any photos or videos of the targets. This gave us a baseline correlation distribution for each neural pattern template. We used the correlation value at the 95th percentile of each distribution as our threshold for correlations during the pre-feedback rest and post-feedback rest. We then correlated the pre-feedback neural pattern with every TR of the pre-feedback rest. We then summed each instance of reactivation within each profile, within each rest, so that for each subject, every profile had a reactivation frequency score for each resting state scan. (We were primarily interested in the percentage increase in reactivation frequency over baseline rest, which was always equivalent to 20, or 5% of the number of TRs in a single resting state scan.)

## Results

### Descriptive statistics and demographics

Each participant provided five different ratings on a 1–9 scale for each of the eight targets, both before receiving feedback and after receiving feedback. Average pre-feedback ratings for each question are as follows: Compatibility: *M* = 4.03, SD = 2.03; Similarity: *M* = 4.12, SD = 1.93; Attractiveness: *M* = 4.68, SD = 2.14; Interest: *M* = 4.97, SD = 1.94; Liking: *M* = 5.08, SD = 1.84. A factor analysis revealed that all five ratings had relatively high loadings onto a single factor (Compatibility: 0.92; Similarity: 0.81; Attractiveness: 0.76; Interest: 0.73; Liking: 0.86). Thus, for subsequent analyses, we used a single “romantic interest” score, computed by taking a weighted average of all five dimensions based on the standardized loadings. In addition, this romantic interest score was binarized when determining whether target feedback was congruent or incongruent with the participant’s initial romantic interest.

After the study, participants were asked several questions to gauge their belief in the cover story. Eighty percent of participants said they wanted to receive the contact information of at least one target, while 70% of participants explicitly said they believed the cover story (although this question was assessed *after* participants were fully debriefed, which may have lowered the number of people who were willing to say they believed the cover story). Behavioral and neural findings are similar for both the full dataset and only those who explicitly believed the cover story, so we report findings for the full dataset for the remainder of the paper.

### How did participants’ romantic interest change in response to social feedback?

To determine how participants’ romantic interest in a target changed in response to social feedback from that target, we first calculated a romantic interest difference score, which was the difference between post-feedback romantic interest and pre-feedback romantic interest. We next ran a model with feedback valence (positive vs negative) and feedback congruence (congruent vs incongruent) as fixed effects and participant as a random effect (since each participant had eight “trials,” or targets).

**Figure 2:**
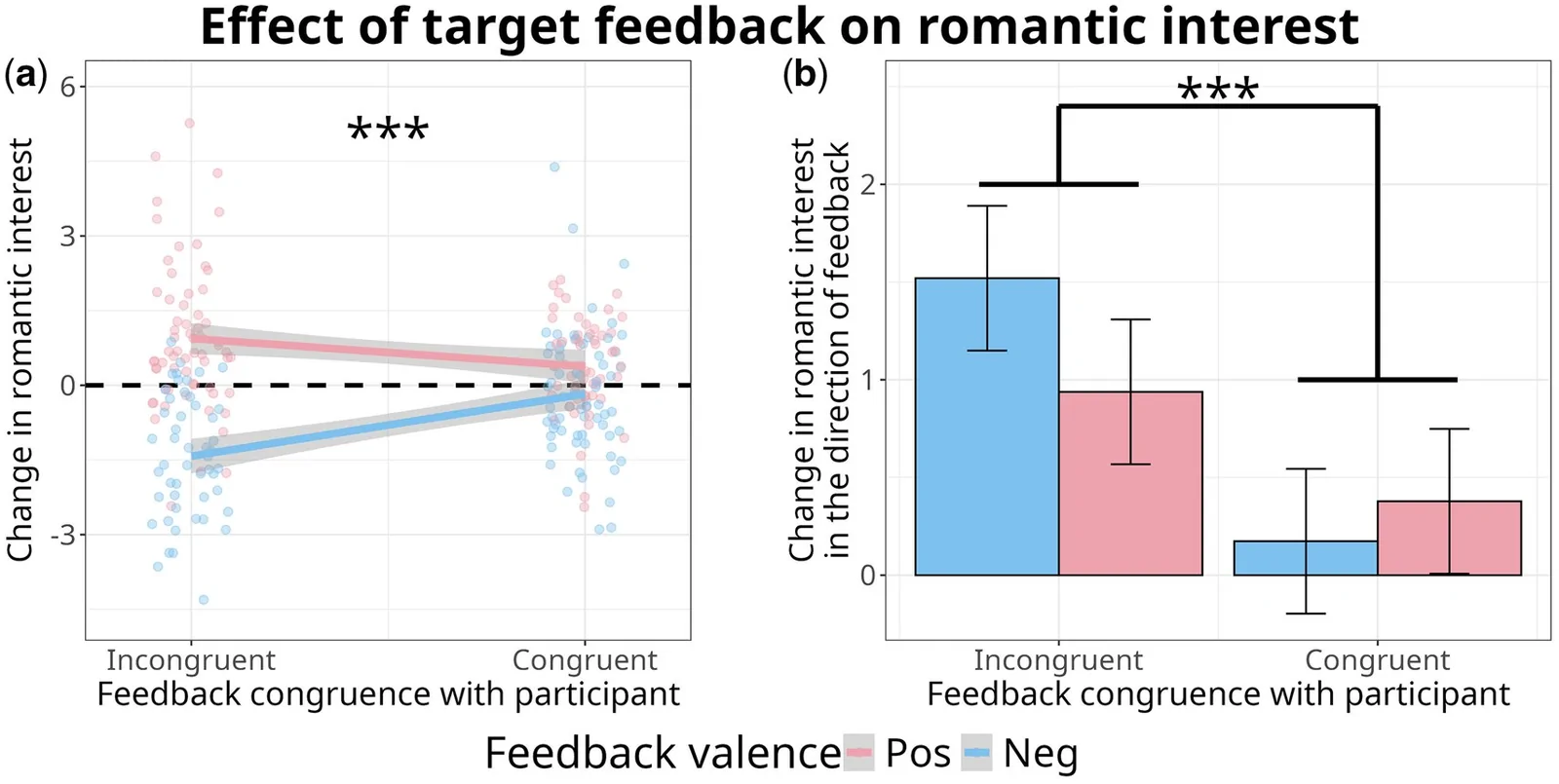
The effect of feedback on romantic interest. (a) Participants increased their ratings in response to incongruent positive feedback and decreased their ratings in response to incongruent negative feedback. (b) Participants’ ratings changed more for incongruent feedback than congruent feedback, but there was no significant difference in the size of the change based on feedback valence (***P < .001, error bars are 95% CIs).

We found a significant effect of feedback valence (*B* = 1.52, SE = 0.21, *P* < .001), where positive feedback led to an increase in romantic interest and negative feedback led to a decrease (Fig. 2a). There was also a significant interaction between feedback valence and feedback congruence (*B* = −2.04, SE = 0.38, *P* < .001), such that the majority of the romantic interest change came from incongruent feedback: There was a significant increase (*M* = 0.938, *t*(63) = 5.11, *P* < .001) in romantic interest for incongruent positive feedback (meaning the participant’s initial romantic interest in the target was low, but the target was romantically interested in the participant) and a significant decrease (*M* = −1.52, *t*(50) = −7.78, *P* < .001) in romantic interest for incongruent negative feedback (meaning the participant’s initial romantic interest in the target was high but the target was not romantically interested in the participant). When the feedback was congruent, there was very little change in romantic interest, regardless of whether the feedback was positive (*M* = 0.38) or negative (*M* = −0.17).

We followed up these results with a model in which we measured how much romantic interest changed in the direction of the feedback (such that increases in romantic interest in response to positive feedback and decreases in romantic interest in response to negative feedback were both measured as positive changes), so we could directly compare the magnitude of the change for positive vs negative feedback (Fig. 2b). We again found larger changes in response to incongruent feedback than congruent feedback (*B* = −1.02, SE = 0.19, *P* < .001), but did not see an effect of feedback valence (*B* = −0.11, SE = 0.20, *P* = .59) or an interaction between the two dimensions of feedback type (*B* = 0.65, SE = 0.45, *P* = .16).

### Did the mentalizing network represent romantic interest?

**Figure 3:**
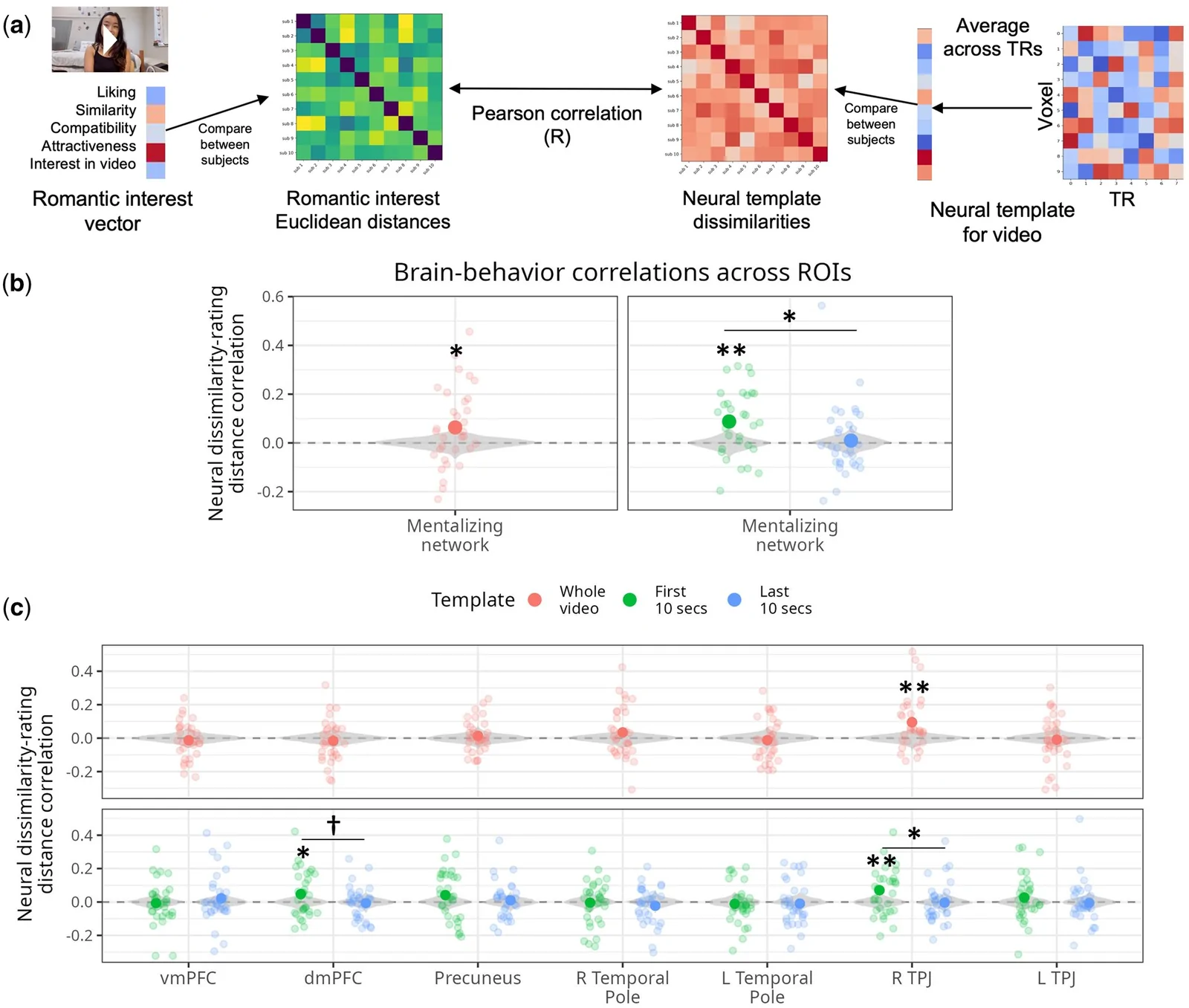
Representational similarity analysis for romantic interest. (a) To conduct an RSA, for each video we created a Euclidean distance matrix, where each cell is the distance between a vector of behavioral ratings, and a neural dissimilarity matrix, where each cell is one minus the Pearson correlation between temporally averaged neural patterns. (Neural patterns were the temporal average of activation patterns for each video.) (b) Comparisons of brain-behavior correlations for each neural pattern, in the mentalizing network. Dots represent correlations averaged across all video matrices, and density plots show the null distribution generated by a permutation test. In the mentalizing network, participants with more similar neural responses to target videos also had more similar behavioral ratings of romantic interest, and this relationship was significantly stronger when using the first 10 s of neural data compared to the last 10 s of neural data for each video. (c) The same visualization for individual mentalizing network ROIs, showing the same pattern of results in right TPJ and a marginally stronger effect in early timepoints for dmPFC (**P < .01; *P < .05; ^†^*P* < .10).

For each video that participants watched, we ran an RSA, whereby we correlated a rating distance matrix (distance here being the Euclidean distance between behavioral rating vectors, where each element of the vector is a rating dimension) and a neural dissimilarity matrix (dissimilarity here being one minus the correlation between the pattern of average activity across all voxels in a particular ROI). If a particular brain area was tracking romantic interest, we would expect to see a positive correlation. A permutation test with 1000 permutations revealed a significant positive correlation across the entire Neurosynth mentalizing network (*r* = 0.063, *P* = .001) (Fig. 3b). Follow-up tests revealed a similar, but stronger, effect specifically for the right TPJ (*r* = 0.095, *P* < .001; Fig. 3c).

**Figure 4:**
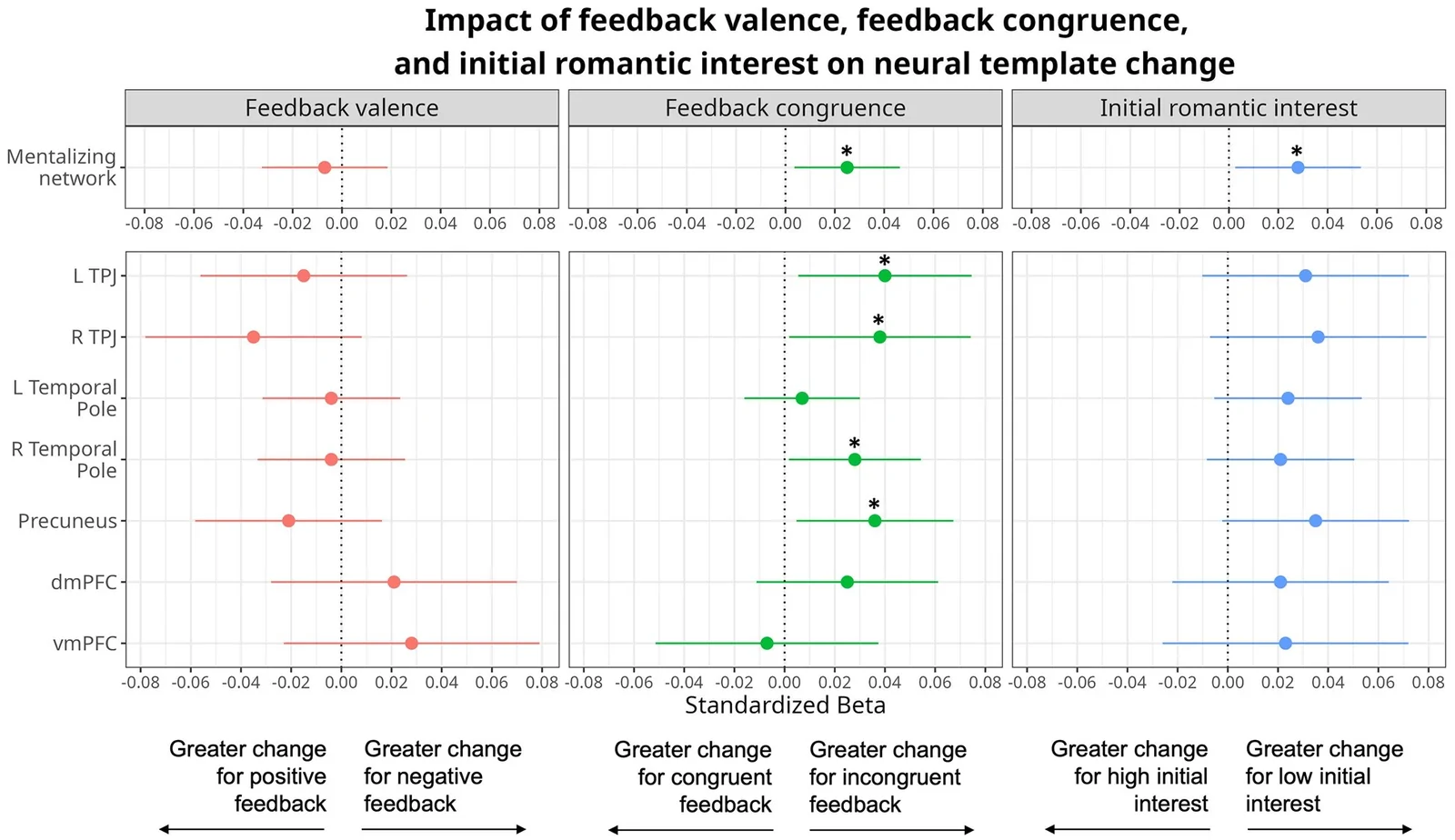
How neural patterns for targets changed between pre- and post-feedback videos. Effects for each ROI for each comparison type on how much neural patterns changed pre-feedback vs post-feedback. (Neural patterns were the temporal average of activation patterns for each video.) There were no significant differences in neural pattern similarity between targets who provided positive feedback and targets who provided negative feedback. One-tailed tests revealed that neural similarity was higher for targets who provided congruent feedback than those who provided incongruent feedback in the mentalizing network, the left and right TPJ, the right temporal pole and the precuneus. For initial romantic interest, pre-post neural similarity was higher in the mentalizing network for high initial interest targets than for low initial interest targets (*P < .05, error bars are 95% CIs for two-tailed tests and 90% CIs for one-tailed tests).

We next wanted to know if the effects described above were driven by neural activity during a particular segment of the video. We conducted identical RSAs, with two different versions of the neural pattern: averaged across the first 10 TRs and averaged across the last 10 TRs. We then compared the strength of the correlations calculated with the first 10 TR pattern to the correlations from the whole-video pattern and the last 10 TR pattern with a permutation test (1000 permutations) that exchanged the two time-window labels within matched observations. We chose to compare these patterns to determine if romantic interest was most strongly represented in the brain in the opening moments of an encounter or at the end of an encounter.

When using the first 10 TRs, we again found significantly positive correlations for the entire mentalizing network (*r* = 0.088, *P* < .001; Fig. 3b) and the right TPJ (*r* = 0.071, *P* = .001; Fig. 3c). We also found a significant correlation for the dmPFC (*r* = 0.047, *P* = .015). When using the last 10 TRs, we found no significant correlations for any of our ROIs. To test the robustness of our results, we also performed RSAs for the first/last 15 s and the first/last 5 s. The results across different time window lengths largely align, and a table with full statistics for these additional analyses can be found in Supplementary Results.

When comparing correlation strength across templates, we found that correlations were significantly stronger with the first 10 TR pattern than with the last 10 TR pattern for the mentalizing network (difference = 0.078, *P* = .011; Fig. 3b) and the right TPJ (difference = 0.074, *P* = .018; Fig. 3c). We also found that correlations were marginally stronger with the first 10 TR pattern than with the whole video pattern for the dmPFC (difference = 0.054, *P* = .069; Fig. 3c).

### How were neural representations of potential romantic partners updated in response to feedback?

Given that the mentalizing network, and several ROIs within that network, appeared to be tracking romantic interest, we next asked if neural representations of targets changed more in response to certain types of feedback—specifically, whether representations changed more in response to incongruent feedback than to congruent feedback. To answer this question, we correlated pre-feedback and post-feedback patterns, for each participant and for each target, and ran a linear regression to compare correlations across each feedback type. Since we had a specific hypothesis about the direction of the effect, we used a one-tailed test of significance, which revealed that the mentalizing network showed greater similarity (in other words, less change) between the pre-feedback and post-feedback patterns when the participant received congruent feedback, as compared to incongruent feedback (*B* = 0.03, SE = 0.01, *P* = .03; Fig. 4). Follow-up tests revealed this effect for several of our other ROIs, including the left TPJ (*B* = 0.04, SE = 0.02 *P* = .04), the right TPJ (*B* = 0.04, SE = 0.02, *P* = .04), the right temporal pole (*B* = 0.03, SE = 0.02, *P* = .04), and the precuneus (*B* = 0.04, SE = 0.02, *P* = .03).

We also compared pre–post similarity based on feedback valence and participants’ initial romantic interest, although we did not have specific hypotheses about the direction of the effect. We found no effects of feedback valence, but found greater similarity (less change) between the two patterns across the whole the mentalizing network when initial romantic interest was high (*B* = 0.03, SE = 0.01, *P* = .03; Fig. 4). This result might suggest that being initially romantically interested in someone is a persistent feature of one’s neural representation of them that is maintained even as the representations change in other ways. It might also be an effect of attention, whereby participants paid greater attention upon a second encounter to targets they were more romantically interested in, leading to a greater neural similarity between the two encounters.

### How often were neural representations of potential romantic partners reactivated during rest in response to feedback?

**Figure 5:**
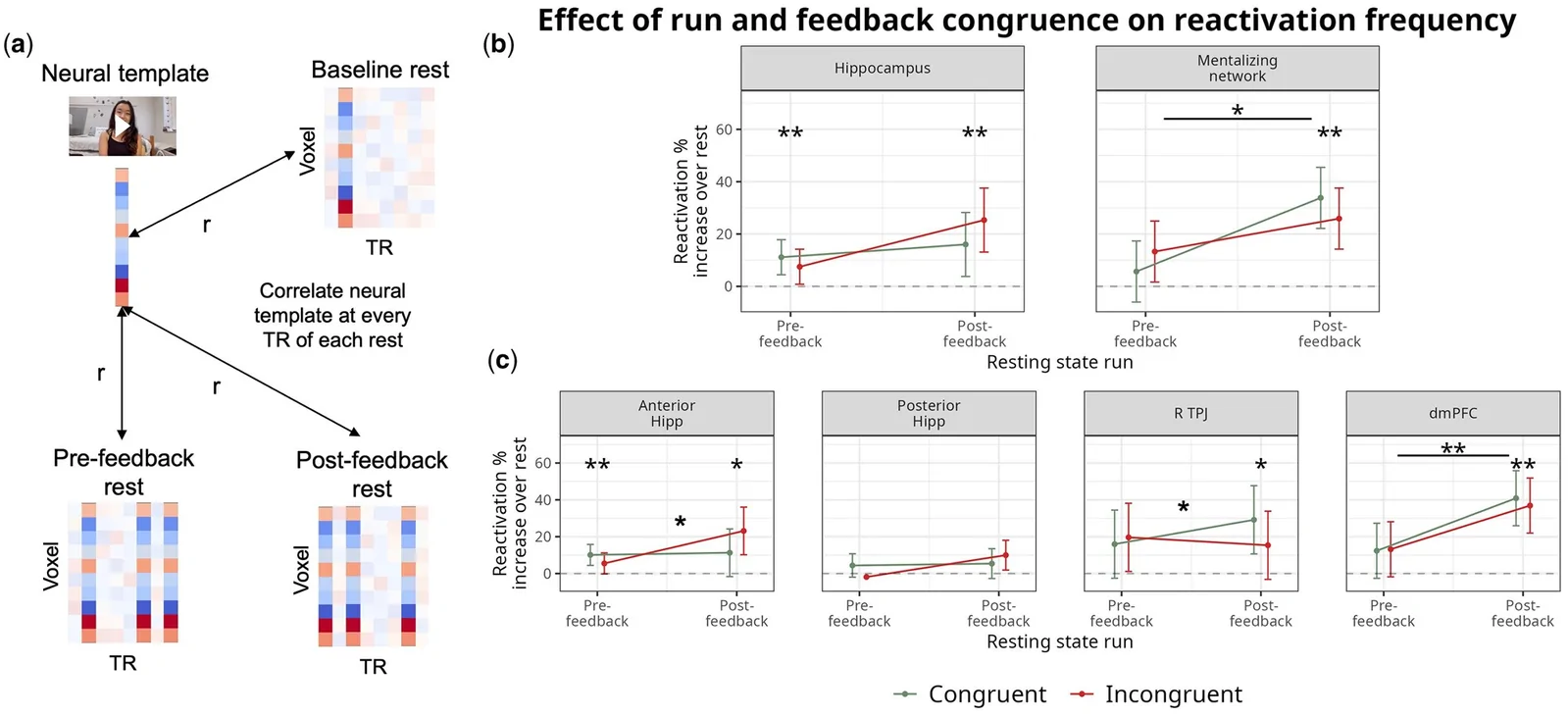
Changes in reactivation frequency during post-encoding rest. (a) We calculated reactivation during post-encoding rest by establishing a reactivation threshold, which was the 95th percentile of a distribution of baseline rest-neural pattern correlation values. Then, we asked how many TRs during pre- and post-feedback rest had correlations that were above that threshold. (b) We visualize reactivation frequency for pre-feedback rest and post-feedback rest as percentage increases over reactivation frequency during baseline rest. The hippocampus demonstrated an increase in reactivation frequency over baseline rest, while the mentalizing network demonstrated an increase in post-feedback rest over pre-feedback rest. (c) Results for select subregions of the hippocampus and the mentalizing network. There was a significant run × feedback congruence interaction in the anterior hippocampus and in the right TPJ (**P < .01; *P < .05).

We next looked to post-encoding rest scans to understand how frequently participants were reactivating targets. More frequent reactivation post-feedback would suggest that the participant finds the feedback meaningful and thinks more about a target in response to receiving feedback from them. In line with our hypothesis, a linear regression model revealed a significant increase in reactivation frequency during post-feedback rest, as compared to pre-feedback rest, across the whole mentalizing network (*B* = 4.16, SE = 1.77, *P* = .03). Follow-up regressions revealed stronger effects for the left temporal pole (*B* = 4.69, SE = 1.46, *P* = .003) and the dmPFC (*B* = 5.25, SE = 1.85, *P* = .008; Fig. 5b and c). None of these ROIs demonstrated a significant increase in reactivation frequency during pre-feedback rest as compared to baseline (mentalizing network: *B* = 1.86, SE = 1.19, *P* = .13; left temporal pole: *B* = 1.25, SE = 1.05, *P* = .24; dmPFC: 2.55, SE = 1.53, *P* = .11), which suggests that reactivation increased in these regions in response to receiving feedback, and not simply in response to an encounter.

We also examined changes in reactivation frequency in the hippocampus, given extensive work that it demonstrates reactivation during post-encoding rest (Schapiro *et al*. 2018, Yu *et al*. 2024). Here, we found an increase in reactivation frequency when comparing pre-feedback rest to baseline (*B* = 1.88, SE = 0.68, *P* = .009), and the effect appeared to be driven by the anterior hippocampus (*B* = 1.58, SE = 0.58, *P* = .009) and not the posterior hippocampus (*B* = 0.28, SE = 0.65, *P* = .67). In addition, we did not observe a significant increase during post-feedback rest as compared to pre-feedback rest (Hippocampus: *B* = 2.20, SE = 1.14, *P* = .06; anterior hippocampus: *B* = 1.80, SE = 1.27, *P* = .17; posterior hippocampus: *B* = 1.23, SE = 0.64, *P* = .07). These results supported our hypothesis that targets were reactivated in the hippocampus after an initial encounter, and were reactivated in the mentalizing network—and more specifically, in the left temporal pole and the dmPFC—after a second encounter and after receiving social feedback.

Finally, we wanted to examine if the increase in reactivation frequency during post-feedback rest was driven by a particular kind of feedback. In a test of feedback valence (positive vs negative), we did not find a significant change in reactivation frequency based on feedback valence in the mentalizing network or in any of our ROIs. However, when we looked at reactivation frequency change as a function of feedback congruence (congruent vs incongruent), we found that incongruent feedback led to a larger increase in reactivation frequency for the anterior hippocampus (*B* = −3.13, SE = 1.34, *P* = .02), while congruent feedback led to a larger increase in reactivation frequency for the right TPJ (*B* = 4.41, SE = 1.75, *P* = .01; Fig. 5b and c). These results suggest the impact of feedback on neural reactivation is dependent on both the dimension of the feedback (valence or congruence) as well as the role of the brain area where it is being reactivated.

## Discussion

In the current study, we set out to investigate three questions about the nature of neural representation of potential romantic partners, and how these representations change over time. We found that the mentalizing network as a whole, and more specifically the dmPFC and the right TPJ, represented romantic interest ratings. We also found that neural representations of targets changed more when the target’s romantic interest was unaligned with the participant’s. We found this effect in the mentalizing network as a whole, as well as in bilateral TPJ, the right temporal pole, and the precuneus. Finally, we found that in the right TPJ, reactivation frequency increased *more* when the target’s romantic interest aligned with the participant’s.

### The mentalizing network represents romantic interest

An RSA showed that target videos eliciting similar patterns of romantic interest in participants were represented more similarly across the entire mentalizing network, with follow-up analyses showing this effect was strongest in the right TPJ. This finding implies that the right TPJ—and to a lesser extent, the mentalizing network as a whole—represents key psychological variables that comprise romantic interest. The TPJ is commonly associated with mentalizing in general (Saxe and Kanwisher 2004, Van Overwalle and Baetens 2009), and previous work using similar multivariate analyses has found that the TPJ tracks others’ mental states (Tamir *et al*. 2016, Thornton *et al*. 2019, Golec-Staśkiewicz *et al*. 2022). Recent work suggests the TPJ may be tracking other types of social evaluation as well, such as trait impressions (Chwe *et al*. 2024), social value (Morelli *et al*. 2018), or personal relevance (Bayer *et al*. 2021). To our knowledge, no other study has demonstrated that the TPJ also represents romantic interest. It may be the case that findings concerning different types of social evaluations in the TPJ, including ours about romantic interest, are united by an attention to the mental states of others. Indeed, the dating context is one in which we are particularly attuned to others’ intentions.

### Neural representations change more when participant–starget romantic interest is unaligned

Participants viewed videos of each target twice: once before receiving feedback, and once after receiving feedback. We found that, within-subject, neural representations changed more in the mentalizing network, the bilateral TPJ, and the precuneus when the target’s romantic interest was unaligned with the participant’s. These results reinforce our behavioral findings, which showed that incongruent feedback led to larger changes in romantic interest ratings. While previous work implicates the TPJ in the process of impression updating (Cloutier *et al*. 2011) and prediction error (Shulman *et al*. 2007, Vetter *et al*. 2011, Dohmatob *et al*. 2020), relatively few studies have demonstrated what we found: neural representations in the TPJ and across the mentalizing network changed more as a result of information that was incongruent with one’s initial beliefs.

These results speak to the fact that romantic interest evaluations are partially based on another person’s evaluation of oneself. Past work on impression updating has been limited to studying inconsistent personality traits (Ferrari *et al*. 2016, Mende-Siedlecki and Todorov 2016), but in everyday life, social evaluations are formed and updated in conjunction with the target’s own evaluations. In addition, we found that neural representations changed according to whether or not the target’s romantic interest was *aligned* with the participants’. This finding emphasizes that we are motivated to be aligned with other people in our relationships, and that impression formation and updating is more accurately studied when the participant has some indication of the target’s impression as well.

In addition, romantic interest evaluations are temporally extended–what happens after an impression update occurs can be just as informative, if not more so, as what happens at the moment that inconsistent information is presented. The idea of representational change is often investigated in the context of memory updating (Zadbood *et al*. 2022, Wahlheim and Zacks 2025), where representations for past events in regions in the default mode network (which has significant overlap with the mentalizing network) change upon the receipt of new information. Here, we show that representations of the same target change according to whether or not information provided by the target aligned with the participants’ initial impression of them.

### Reactivation frequency increases in the mentalizing network in response to feedback and in the TPJ in response to congruent feedback

How one thinks about another person is not the only thing that might change after an impression update occurs—how often one thinks about them might change as well, as people tend to think more about things they find more rewarding (Murty and Adcock 2014, Gruber *et al*. 2016), especially social information (Meyer *et al*. 2019, Jimenez and Meyer 2024). Consistent with previous work on hippocampal reactivation (Staresina *et al*. 2012, Gruber *et al*. 2016, Schapiro *et al*. 2018, Tambini and Davachi 2019, Yu *et al*. 2024), we found that reactivation frequency increased in the hippocampus when compared with a baseline rest before participants had seen any target videos.

When comparing post-feedback rest to pre-feedback rest, however, we observed a significant increase in reactivation frequency in the mentalizing network—and specifically, the dmPFC and the left temporal pole—rather than the hippocampus. Previous work has demonstrated that these brain areas, and areas across the mentalizing network, prioritize social information during post-encoding rest (Meyer *et al*. 2019, Jimenez and Meyer 2024), so it makes sense that they would demonstrate an increase in reactivation in response to learning about socially relevant information (i.e. finding out the target’s romantic interest in oneself).

What impacted reactivation frequency most was whether or not the target’s romantic interest was aligned with the participant’s. Specifically, we found that in the anterior hippocampus, reactivation increased more for *incongruent* feedback, which is perhaps evidence that the feedback is being treated as an expectancy violation (Somerville *et al*. 2006, 2010) or schema-inconsistent information (van Kesteren *et al*. 2013, Bein *et al*. 2014, Varga *et al*. 2025). Most of this work demonstrates an increase in hippocampal activity upon receipt of the information or during consolidation. Our study extends these findings by showing that incongruent information leads to increased hippocampal reactivation for stimuli associated with that incongruent information.

On the flip side, we found that reactivation frequency increased more in the right TPJ in response to congruent feedback than it did to incongruent feedback. In essence, participants thought more about targets whose romantic interest levels aligned with the participants’, regardless of if the target was or was not romantically interested in the participant. Our study is the first to investigate reactivation in the context of impression updating, which may be why previous studies have not found reactivation effects in the TPJ.

Although these results were at first surprising, they make sense in light of our findings regarding changes in neural representations, and in light of the fact that people are motivated to view the world similarly to others, especially for a high-stakes evaluation like romantic interest. In our study, people changed their perceptions of those they *were not* aligned with—because they were motivated to have their views match those of others—and thought more about those they *were* aligned with—because it was rewarding to think about those they were already aligned with. In both cases, we see evidence that alignment with others is a motivating force. Indeed, for the latter finding, we see that effect regardless of if the alignment is around mutual romantic interest or mutual romantic disinterest, which we interpret as evidence of the salience of alignment as a relevant factor in social relationships. Quite simply, it is reassuring (and motivating) to know that your belief about someone matches their belief about you. These results demonstrate that impression formation and updating do not occur in isolation from the evaluations of others.

There are several limitations of this study, as well as opportunities for future research. First, we conducted RSA between-subjects rather than within-subjects, which may limit the inferences we can draw from our findings. We set up our RSA in this way because we wanted to hold the stimuli constant so that we could ensure differences in representations were driven by idiosyncratic social impressions and not low-level sensory features. Second, each participant had two runs, and the relationship between neural activity and romantic interest ratings may have differed between them. Between-subjects RSA at the item level ensured more consistency in our interpretation. Finally, our permutation tests occur at the participant level, which strengthens our ability to generalize our results beyond our sample. One avenue for future research that makes use of between-subjects RSA would be a study that more closely examines the temporal dynamics of impression updating. Our analytic approach did not preserve the temporal dimension of our stimulus set (although we do include an exploratory analysis of this kind in Supplementary Results), which could speak to how impressions correspond to the emergence over time of specific information from the target. A future study can use this information to investigate temporal intersubject correlations—in essence, how idiosyncratically participants’ impressions unfold.

Second, the structure of our study—and the timing of the receipt of feedback—means that feedback is confounded with the presentation of additional information about the target in the form of a second video. Thus, it is difficult to say with certainty that any effects we see after the second video are the result of feedback, and not simply the new information presented in the second video. However, we believe this explanation is unlikely for two reasons. First, the second video—both within and across targets—is counterbalanced across subjects, meaning that the exact information presented post-feedback is different. Second, we saw different patterns of changes in reactivation frequency across brain regions, rather than a uniform increase after each set of videos, which suggests that the presentation of additional information is not the driving factor in our results.

Third, one fifth of participants indicated a level of disinterest in the dating aspect of the task during a debriefing, which may have affected the magnitude of the effects we found. We conducted several behavioral pilot studies in order to refine our cover story and paradigm and maximize its believability, but it can be difficult to achieve 100% believability in a complex social relationships task. Even so, behavioral and neural findings for those that did not believe the cover story were not meaningfully different from those that did, and our study still goes further in terms of ecological validity than many other social neuroscience studies that rely on hypothetical situations.

Finally, future studies may wish to examine individual differences that moderate these findings. These individual differences may include trait-level psychological dimensions, such as self-esteem or rejection sensitivity, as we might predict that participants more sensitive to social cues may show larger differences in neural representations after receiving feedback. Individual differences may also include differences based on gender, sexuality, and race, which we did not test for here, partially because of our limited sample size. Crucially, our sample (as well as our stimulus set) was not limited to one particular gender, sexuality, or race, even though those factors are often relevant in a dating context, because we wanted to maximize the generalizability of our findings.

In summary, we demonstrated in the current study that mentalizing plays a crucial role in forming and updating romantic interest evaluations for potential romantic partners. An RSA revealed that the mentalizing network—and specifically, the right TPJ and the dmPFC—tracked romantic interest over the course of an entire encounter. In addition, after a potential romantic partner provided their own romantic interest, we observed two changes in the mentalizing network that speak to both the socially dependent and the temporally extended nature of impression formation. Specifically, we found that the structure of neural representations changed more when a potential partner’s romantic interest was *unaligned* with the participant’s, but that the frequency with which these neural representations were reactivated increased more when a potential partner’s romantic interest was *aligned* with the participant’s. Both findings demonstrate that people find alignment with others motivating: People change their views of others when those views *don’t match* how others view them, and they think more about others when their views *do match* how others view them. Thus, our study emphasizes that impressions of others are updated in response to motivationally relevant feedback, and that this information needs to be accounted for in order to accurately understand how relationships form and change in everyday life.

## Supplementary Material

nsag074_Supplementary_Data

## Data Availability

Data and scripts for all analyses, as well as detailed information about stimuli, can be found on the project’s OSF page. More information about analysis pipelines can also be found on the project’s Github page.
