# Supplementary material for: The mentalizing network updates neural representations of romantic interest in response to social feedback
Source: Soc Cogn Affect Neurosci. 2026 Sep 4;21(1):nsag074. doi: 10.1093/scan/nsag074 (PMC13597127; doi:10.1093/scan/nsag074)
Supplement: Supplementary file 1 [file nsag074_supplementary_data.docx]

**Supplemental materials**

**Supplemental Methods**

*Video creation*

We hired 16 actors (8 men and 8 women) to make two videos each, for a total of 32 videos in the stimulus set. Each actor was sent two lists of three prompts that are commonly found on dating profiles, such as: What do you like to do in your free time? What are your goals for the future? (A full list of prompts for each video can be found on the OSF page (https://osf.io/qzx3b/?view_only).) Each actor was instructed to film the videos in a quiet room and to speak directly to the camera. They were told to speak in response to each prompt for roughly 30 seconds, and that each video should be in total between 80 and 100 seconds. They were told to speak truthfully about themselves, but to not discuss where they currently lived or their political views to ensure that those were not factors in participants’ romantic interest. Videos were normed for physical attractiveness, perceived age, and perceived sexuality.

*Target/video selection*

While we used eight male targets and eight female targets in the study, we initially sought to hire ten actors of each gender, to give us 40 videos total. We ended up with videos from 9 men and 10 women due to the failure of one male actor to send in their videos, giving us 38 videos total.

After all videos were created, we recruited 53 participants via Prolific to provide ratings on the videos to ensure that the videos were perceived similarly on relevant dimensions, including physical attractiveness, perceived age, and perceived sexuality.

First, we did not want any targets who were outliers on physical attractiveness, to ensure that that target’s videos were not treated categorically differently across our participants. Attractiveness ratings were on a 0-100 scale. Mean attractiveness for female targets was 66.8 (SD = 13.2, range = 38.7-85.4) and mean attractiveness for male targets was 58.3 (SD = 12.2, range = 46.9-74.7). Using visual inspection of the distribution of average attractiveness ratings across targets for each gender, as well as Tukey’s method, which excludes datapoints that are above or below 1.5 times the interquartile range, we excluded one female target from the stimulus set (IQR = 60.8-74.3, mean attractiveness rating for excluded target = 38.7).

Second, we wanted to ensure that the targets appeared to be in the same age range as our participants (18-29). Mean perceived age for female targets was 23.6 (SD = 1.86, range = 20.9-26.3). Mean perceived age for male targets was 25.5 (SD = 1.97, range = 23.4-29.1). No targets had a perceived age range outside of our participants’ age range, so no targets were excluded based on this metric.

Finally, we did not want to exclude participants from our main study based on their sexuality. In order to ensure that targets would be treated similarly by participants regardless of whether the participant who was viewing them had the same gender or a different gender as the target, we needed to ensure that the target did not clearly “present” as one sexuality over another. To do this, we asked our Prolific participants how they “read” the target’s sexuality on a 0-100 scale, ranging from “Definitely straight” to “Definitely queer,” where queer was defined as “For the purposes of this question, ‘queer’ encompasses any sexuality that is not heterosexual, i.e. gay, bi, pan, asexual, etc.” Ideally, Prolific participants would view the targets as equally likely as being straight or queer, meaning the target’s average perceived sexuality score would be close to 50. Mean perceived sexuality scores were quite close to 50 (Female targets: M = 46.2, SD = 17.6; Male targets: M = 43.4, SD = 15.1). We decided that we would exclude targets who had average perceived sexuality scores above 75 or below 25. These criteria led to our excluding one male target (whose score was 15.9) and one female target (whose score was 83.7).

*Additional scanner procedures*

Before viewing each set of target videos, participants completed a photo-viewing scan, which involved viewing photos of each of the targets whose videos they would be viewing during the scan. Each photo was presented for three seconds and was separated by a jittered inter-trial interval, between 1.5 and 5.5 seconds. A photo of each of the eight targets was presented four times, for a total of 32 photo presentations. The order of the photo presentations was randomized. Data from the photo-viewing scan is not analyzed in this paper.

After each set of videos, participants completed a cued-recall task while in the fMRI scanner. Participants were cued with the name and photo of a target and were told to say out loud everything they could remember from that target’s video. After participants finished speaking, they moved on to the next target. The results from the cued-recall task are not discussed in this paper.

*Romantic interest calculation*

Each participant provided five different ratings on a 1-9 scale (Compatibility: “How romantically compatible do you believe you are with this person?” Similarity: “How similar do you believe you are to this person?” Attractiveness: “How physically attractive do you find this person?” Interest: “How interested were you in what this person was talking about?” Liking: “How much do you like this person?”) for each of the eight targets, both before receiving feedback and after receiving feedback. Average pre-feedback ratings for each question are as follows: Compatibility: M = 4.03, SD = 2.03; Similarity: M = 4.12, SD = 1.93; Attractiveness: M = 4.68, SD = 2.14; Interest: M = 4.97, SD = 1.94; Liking: M = 5.08, SD = 1.84. A factor analysis revealed that all five ratings had relatively high loadings onto a single factor (Compatibility: 0.92; Similarity: 0.81; Attractiveness: 0.76; Interest: 0.73; Liking: 0.86). Thus, for subsequent analyses we used a single “romantic interest” score, computed by taking a weighted average of all five dimensions based on the standardized loadings.

*fMRIPrep preprocessing*

Anatomical data preprocessing: The T1-weighted (T1w) image was corrected for intensity non-uniformity (INU) with N4BiasFieldCorrection (Tustison et al. 2010), distributed with ANTs 2.3.3 (Avants et al. 2008), and used as T1w-reference throughout the workflow. The T1w-reference was then skull-stripped with a *Nipype* implementation of the antsBrainExtraction.sh workflow, using OASIS30ANTs as target template. Brain tissue segmentation of cerebrospinal fluid (CSF), white-matter (WM) and gray-matter (GM) was performed on the brain-extracted T1w using fast (FSL 5.0.9, Zhang, Brady, and Smith 2001). Brain surfaces were reconstructed using recon-all (FreeSurfer 6.0.1, Dale, Fischl, and Sereno 1999), and the brain mask estimated previously was refined with a custom variation of the method to reconcile ANTs-derived and FreeSurfer-derived segmentations of the cortical gray-matter of Mindboggle (Klein et al. 2017). Volume-based spatial normalization to one standard space was performed through nonlinear registration with antsRegistration (ANTs 2.3.3), using brain-extracted versions of both T1w reference and the T1w template.

Functional data preprocessing: First, a reference volume and its skull-stripped version were generated. Susceptibility distortion correction was omitted. The BOLD reference was then co-registered to the T1w reference using bbregister (FreeSurfer) which implements boundary-based registration (Greve and Fischl 2009). Co-registration was configured with six degrees of freedom. Head-motion parameters with respect to the BOLD reference (transformation matrices, and six corresponding rotation and translation parameters) are estimated before any spatiotemporal filtering using mcflirt (FSL 5.0.9, Jenkinson et al. 2002). BOLD runs were slice-time corrected to 0.46s (0.5 of slice acquisition range 0s-0.92s) using 3dTshift from AFNI (Cox and Hyde 1997).

The BOLD time-series were resampled onto fsaverage6, and then resampled onto their original, native space by applying the transforms to correct for head-motion. Several confounding time-series were calculated based on the preprocessed BOLD: framewise displacement (FD), DVARS and three region-wise global signals. FD was computed using two formulations following Power (absolute sum of relative motions, Power et al. (2014)) and Jenkinson (relative root mean square displacement between affines, Jenkinson et al. (2002)). FD and DVARS were calculated for each functional run, both using their implementations in *Nipype* (following the definitions by Power et al. 2014). The three global signals were extracted within the CSF, the WM, and the whole-brain masks. Additionally, a set of physiological regressors were extracted to allow for component-based noise correction (CompCor, Behzadi et al. 2007).

Principal components were estimated after high-pass filtering the preprocessed BOLD time-series (using a discrete cosine filter with 128s cut-off) for the two CompCor variants: temporal (tCompCor) and anatomical (aCompCor). tCompCor components were then calculated from the top 2% variable voxels within the brain mask. For aCompCor, three probabilistic masks (CSF, WM and combined CSF+WM) were generated in anatomical space. This mask is obtained by dilating a GM mask extracted from the FreeSurfer’s *aseg* segmentation, and it ensures components are not extracted from voxels containing a minimal fraction of GM. Finally, these masks are resampled into BOLD space and binarized by thresholding at 0.99. For each CompCor decomposition, the *k* components with the largest singular values are retained, such that the retained components’ time series are sufficient to explain 50 percent of variance across the nuisance mask (CSF, WM, combined, or temporal). The remaining components are dropped from consideration.

The confound time series derived from head motion estimates and global signals were expanded with the inclusion of temporal derivatives and quadratic terms for each (Satterthwaite et al. 2013). Frames that exceeded a threshold of 0.5 mm FD or 1.5 standardized DVARS were annotated as motion outliers. All resamplings can be performed with a single interpolation step by composing all the pertinent transformations (i.e. head-motion transform matrices, susceptibility distortion correction when available, and co-registrations to anatomical and output spaces). Gridded (volumetric) resamplings were performed using antsApplyTransforms (ANTs), configured with Lanczos interpolation to minimize the smoothing effects of other kernels (Lanczos 1964). Non-gridded (surface) resamplings were performed using mri_vol2surf (FreeSurfer).

**Supplemental Results**

*RSA with beginning vs end of videos*

We reran our video-segment RSA – where we correlated romantic interest ratings with neural data from just the first 10 seconds and just the last 10 seconds of each video – using the first 5 seconds and the last 5 seconds, as well as the first 15 seconds and the last 15 seconds of each video. Results for these analyses can be found in *Supplemental Table 2*. In sum, our beginning vs end results – whereby the RSA was stronger in the beginning of the video than the end across multiple mentalizing network regions – are largely consistent, regardless of the length of the beginning segment and the end segment used in the analysis.

| **ROI** | **Segment** | **Time window** | **Observed cor** | **p** |
| --- | --- | --- | --- | --- |
| Mentalizing network | Beginning | First 15 seconds† | 0.033 | 0.06 |
|  |  | First 5 seconds* | 0.060 | 0.003 |
|  |  | First 10 seconds* | 0.088 | < 0.001 |
|  | End | Last 15 seconds | 0.016 | 0.21 |
|  |  | Last 5 seconds | 0.011 | 0.31 |
|  |  | Last 10 seconds | 0.010 | 0.32 |
| rTPJ | Beginning | First 15 seconds* | 0.044 | 0.018 |
|  |  | First 5 seconds | 0.019 | 0.19 |
|  |  | First 10 seconds* | 0.071 | 0.001 |
|  | End | Last 15 seconds | -0.002 | 0.54 |
|  |  | Last 5 seconds | -0.011 | 0.69 |
|  |  | Last 10 seconds | -0.003 | 0.57 |
| dmPFC | Beginning | First 15 seconds | 0.014 | 0.23 |
|  |  | First 5 seconds | 0.025 | 0.11 |
|  |  | First 10 seconds* | 0.047 | 0.015 |
|  | End | Last 15 seconds | 0.001 | 0.47 |
|  |  | Last 5 seconds | 0.024 | 0.13 |
|  |  | Last 10 seconds | -0.007 | 0.62 |

*Supplemental Table 1.*

*Temporal IS-RSA*

In addition to the spatial IS-RSA reported in the main text – for which we averaged our neural data over time to create “neural templates” for each video for each participant – we also conducted an IS-RSA that averaged data over the spatial dimension to provide a measure of average neural activity in a particular brain region at each timepoint during the video. Our primary analysis in the main text ignored the temporal dimensions of the video, instead relying on the video stimuli as a way to increase the robustness of our measures and the attention of our participants. That analysis allowed us to ask how our overall impressions of another person changed in response to social feedback. On the other hand, the current analysis allowed us to evaluate online impression formation and updating over a short timescale, and not in response to feedback. Essentially, it asks whether participants who have neural signals with similar trajectories while watching a video end up with similar behavioral ratings of social impressions.

This temporally-focused analysis did not yield a significant brain-behavior relationship in any of our ROIs (See *Supplemental Figure 1*.) For the mentalizing network as a whole, the observed average brain-behavior correlation was -0.003 (p = 0.57).

*Supplemental Figure 1.*

*Representational change*

The main text of our paper reports differences in within-profile representational change between different feedback types (positive vs negative, congruent vs incongruent) and different initial romantic interest (high vs low).

In *Supplemental Figure 2*, we report average pre-post correlations for all feedback types. All correlations are relatively low due to the complexity of the representations in these brain areas, which is why in the main text we prioritized analyses that focus on the correlation difference between feedback types. We found that pre-post within-subject similarity for neural representations for targets who provide congruent feedback is significantly above 0 in the mentalizing network as a whole (B = 0.023, SE = 0.009, p = 0.024) and the left TPJ (B = 0.035, SE = 0.014, p = 0.015). We also found that pre-post similarity was higher for targets in whom participants had high initial romantic interest in the same two ROIs (mentalizing network: B = 0.027, SE = 0.01, p = 0.016; left TPJ: B = 0.033, SE = 0.016, p = 0.033).

**Supplemental References**

Abraham, Alexandre, Fabian Pedregosa, Michael Eickenberg, Philippe Gervais, Andreas Mueller, Jean Kossaifi, Alexandre Gramfort, Bertrand Thirion, and Gael Varoquaux. 2014. “Machine Learning for Neuroimaging with Scikit-Learn.” *Frontiers in Neuroinformatics* 8. <https://doi.org/10.3389/fninf.2014.00014>.

Avants, B.B., C.L. Epstein, M. Grossman, and J.C. Gee. 2008. “Symmetric Diffeomorphic Image Registration with Cross-Correlation: Evaluating Automated Labeling of Elderly and Neurodegenerative Brain.” *Medical Image Analysis* 12 (1): 26–41. <https://doi.org/10.1016/j.media.2007.06.004>.

Behzadi, Yashar, Khaled Restom, Joy Liau, and Thomas T. Liu. 2007. “A Component Based Noise Correction Method (CompCor) for BOLD and Perfusion Based fMRI.” *NeuroImage* 37 (1): 90–101. <https://doi.org/10.1016/j.neuroimage.2007.04.042>.

Cox, Robert W., and James S. Hyde. 1997. “Software Tools for Analysis and Visualization of fMRI Data.” *NMR in Biomedicine* 10 (4-5): 171–78. [https://doi.org/10.1002/(SICI)1099-1492(199706/08)10:4/5<171::AID-NBM453>3.0.CO;2-L](https://doi.org/10.1002/(SICI)1099-1492(199706/08)10:4/5%3c171::AID-NBM453%3e3.0.CO;2-L).

Dale, Anders M., Bruce Fischl, and Martin I. Sereno. 1999. “Cortical Surface-Based Analysis: I. Segmentation and Surface Reconstruction.” *NeuroImage* 9 (2): 179–94. <https://doi.org/10.1006/nimg.1998.0395>.

Esteban, Oscar, Ross Blair, Christopher J. Markiewicz, Shoshana L. Berleant, Craig Moodie, Feilong Ma, Ayse Ilkay Isik, et al. 2018. “FMRIPrep.” *Software*. Zenodo. <https://doi.org/10.5281/zenodo.852659>.

Esteban, Oscar, Christopher Markiewicz, Ross W Blair, Craig Moodie, Ayse Ilkay Isik, Asier Erramuzpe Aliaga, James Kent, et al. 2018. “fMRIPrep: A Robust Preprocessing Pipeline for Functional MRI.” *Nature Methods*. <https://doi.org/10.1038/s41592-018-0235-4>.

Fonov, VS, AC Evans, RC McKinstry, CR Almli, and DL Collins. 2009. “Unbiased Nonlinear Average Age-Appropriate Brain Templates from Birth to Adulthood.” *NeuroImage* 47, Supplement 1: S102. <https://doi.org/10.1016/S1053-8119(09)70884-5>.

Gorgolewski, K., C. D. Burns, C. Madison, D. Clark, Y. O. Halchenko, M. L. Waskom, and S. Ghosh. 2011. “Nipype: A Flexible, Lightweight and Extensible Neuroimaging Data Processing Framework in Python.” *Frontiers in Neuroinformatics* 5: 13. <https://doi.org/10.3389/fninf.2011.00013>.

Gorgolewski, Krzysztof J., Oscar Esteban, Christopher J. Markiewicz, Erik Ziegler, David Gage Ellis, Michael Philipp Notter, Dorota Jarecka, et al. 2018. “Nipype.” *Software*. Zenodo. <https://doi.org/10.5281/zenodo.596855>.

Greve, Douglas N, and Bruce Fischl. 2009. “Accurate and Robust Brain Image Alignment Using Boundary-Based Registration.” *NeuroImage* 48 (1): 63–72. <https://doi.org/10.1016/j.neuroimage.2009.06.060>.

Jenkinson, Mark, Peter Bannister, Michael Brady, and Stephen Smith. 2002. “Improved Optimization for the Robust and Accurate Linear Registration and Motion Correction of Brain Images.” *NeuroImage* 17 (2): 825–41. <https://doi.org/10.1006/nimg.2002.1132>.

Klein, Arno, Satrajit S. Ghosh, Forrest S. Bao, Joachim Giard, Yrjö Häme, Eliezer Stavsky, Noah Lee, et al. 2017. “Mindboggling Morphometry of Human Brains.” *PLOS Computational Biology* 13 (2): e1005350. <https://doi.org/10.1371/journal.pcbi.1005350>.

Lanczos, C. 1964. “Evaluation of Noisy Data.” *Journal of the Society for Industrial and Applied Mathematics Series B Numerical Analysis* 1 (1): 76–85. <https://doi.org/10.1137/0701007>.

Power, Jonathan D., Anish Mitra, Timothy O. Laumann, Abraham Z. Snyder, Bradley L. Schlaggar, and Steven E. Petersen. 2014. “Methods to Detect, Characterize, and Remove Motion Artifact in Resting State fMRI.” *NeuroImage* 84 (Supplement C): 320–41. <https://doi.org/10.1016/j.neuroimage.2013.08.048>.

Satterthwaite, Theodore D., Mark A. Elliott, Raphael T. Gerraty, Kosha Ruparel, James Loughead, Monica E. Calkins, Simon B. Eickhoff, et al. 2013. “An improved framework for confound regression and filtering for control of motion artifact in the preprocessing of resting-state functional connectivity data.” *NeuroImage* 64 (1): 240–56. <https://doi.org/10.1016/j.neuroimage.2012.08.052>.

Tustison, N. J., B. B. Avants, P. A. Cook, Y. Zheng, A. Egan, P. A. Yushkevich, and J. C. Gee. 2010. “N4ITK: Improved N3 Bias Correction.” *IEEE Transactions on Medical Imaging* 29 (6): 1310–20. <https://doi.org/10.1109/TMI.2010.2046908>.

Zhang, Y., M. Brady, and S. Smith. 2001. “Segmentation of Brain MR Images Through a Hidden Markov Random Field Model and the Expectation-Maximization Algorithm.” *IEEE Transactions on Medical Imaging* 20 (1): 45–57. <https://doi.org/10.1109/42.906424>.
